# Parameters of Oxidative Stress, Vitamin D, Osteopontin, and Melatonin in Patients with Lip, Oral Cavity, and Pharyngeal Cancer

**DOI:** 10.1155/2021/2364931

**Published:** 2021-10-20

**Authors:** Jarosław Nuszkiewicz, Jolanta Czuczejko, Marta Maruszak, Marta Pawłowska, Alina Woźniak, Bogdan Małkowski, Karolina Szewczyk-Golec

**Affiliations:** ^1^Department of Medical Biology and Biochemistry, Faculty of Medicine, Ludwik Rydygier Collegium Medicum in Bydgoszcz, Nicolaus Copernicus University in Toruń, 24 Karłowicza St., 85-092 Bydgoszcz, Poland; ^2^Department of Psychiatry, Faculty of Medicine, Ludwik Rydygier Collegium Medicum in Bydgoszcz, Nicolaus Copernicus University in Toruń, 9 M. Curie Skłodowskiej St., 85-094 Bydgoszcz, Poland; ^3^Department of Nuclear Medicine, Oncology Centre Prof, Franciszek Łukaszczyk Memorial Hospital, Bydgoszcz, 2 Dr I. Romanowskiej St., 85-796 Bydgoszcz, Poland; ^4^Department of Diagnostic Imaging, Faculty of Health Sciences, Ludwik Rydygier Collegium Medicum in Bydgoszcz, Nicolaus Copernicus University in Toruń, 2 Dr I. Romanowskiej St., 85-796 Bydgoszcz, Poland

## Abstract

Lip, oral cavity, and pharyngeal cancers (LOCP) constitute a group of rare neoplasms with unfavorable prognosis. So far, not much is known about the role of vitamin D and oxidative stress in the pathogenesis of LOCP in the European population. The aim of the study was to determine the concentrations of vitamin D, osteopontin, melatonin, and malondialdehyde (MDA) as markers of oxidative stress and/or inflammation, as well as the activities of antioxidant enzymes in the course of LOCP. The vitamin D, melatonin, and osteopontin concentrations in blood serum, the MDA levels in erythrocytes and blood plasma, and the activities of superoxide dismutase (SOD-1), catalase (CAT), and glutathione peroxidase (GPx) in erythrocytes were measured in blood samples taken from 25 LOCP patients of middle age (YCG), 20 LOCP elderly patients (OCG), and 25 healthy middle-aged volunteers. In both cancer groups, decreases in vitamin D and CAT, as well as increases in osteopontin and blood plasma MDA, were observed. An increase in GPx activity in YCG and a decrease in melatonin level in OCG were found. The results indicate the vitamin D deficiency and disturbed oxidant-antioxidant homeostasis in LOCP patients. Osteopontin seems to be associated with LOCP carcinogenesis and requires further research.

## 1. Introduction

Lip, oral cavity, and pharyngeal cancers (LOCP) belong to the most common head and neck cancers worldwide. Moreover, scientific analyses indicate that the incidence of this type of neoplasm will increase in the future. According to data, in 2012, 529,500 new cases of LOCP were detected worldwide, which corresponds to 3.6% of all cancers [1, 2]. Mortality in 2012 from this group of neoplasms was estimated at 292,300 cases, which corresponds to 3.6% of deaths due to neoplastic diseases [1, 2]. Projections for 2035 show a 62% increase in the number of cases to around 856,000 cases annually [1]. Cancers of lip, oral cavity, and pharynx are considered together because they are characterized by similar risk factors. Neoplasms belonging to this group affect male much more often than female, and the age group 50-70 years is particularly vulnerable [3–5]. In addition, this type of cancer is especially common in south-central Asia [1]. The main risk factors for the development of LOCP cancer include smoking [6], alcohol consumption [7, 8], infections caused by Epstein-Barr virus (EBV) [9], and *human papillomavirus* (HPV) [10]. Early diagnosis and treatment initiation significantly increases patient survival; unfortunately, most cases are detected in the advanced stage of the disease, which lowers the 5-year survival rate to about 40% [3].

Pathogenesis of lip, oral cavity, and pharyngeal cancers is still not clear and is believed to be multifactorial in origin. Few studies indicate the participation of extracellular matrix and fibroblast changes, immune system, and oxidative stress in the pathogenesis of oral submucous fibrosis, leading to cancer of the oral cavity [11]. Additionally, molecular pathogenesis of head and neck cancer is associated with deletion in region located at chromosome 9p21–22 containing p16 tumor suppressor gene [12]. An inherent element of carcinogenesis and neoplastic disease is the increased generation of reactive oxygen species (ROS) [13–18]. Moreover, cancer cells synthesize and secrete cytokines that modulate inflammation and significantly increases ROS generation [19]. The disturbance of homeostasis by ROS generated in exceeding of physiological capacity of adaptation leads to oxidative stress [20–22]. Although the disease has a specific localization, systemic symptoms of oxidative stress are observed in patients [23, 24]. ROS are a group of chemical molecules which are characterized by the presence of nonpair electrons and high chemical reactivity [25]. The most important ROS include superoxide anion (O_2_^−^), hydrogen peroxide (H_2_O_2_), hydroxyl radical (OH^−^), and singlet oxygen (^1^O_2_) [20, 25]. Due to high chemical reactivity, ROS modify proteins, lipids, and genetic material [26, 27]. The effect of lipid peroxidation is damage to cell membranes, and the main markers of this process is malondialdehyde (MDA) and 4-hydroxynonenal [28]. Antioxidants play an important role in maintaining the redox balance [29]. Endogenous antioxidants include enzymes such as superoxide dismutases (SODs), catalase (CAT), and glutathione peroxidases (GPxs) [30]. The antioxidant defense is also constituted by small endo- and exogenous biomolecules such as vitamins A, C, and E, melatonin, and glutathione (GSH) [31, 32]. The role of vitamin D as an antioxidant remains ambiguous due to inconclusive research results [33].

Vitamin D is a biomolecule with pleiotropic properties. Calcitriol (1,25-dihydroxycholecalciferol) plays the most important role among the group of compounds called vitamin D [34]. Chemical compounds belonging to this group can be absorbed with food, most often in the form of cholecalciferol and ergocalciferol [35, 36]. Another source of vitamin D is the endogenous synthesis under the influence of ultraviolet radiation (UV) and hydroxylases found in the liver and kidney. The substrate for this process is 7-dehydrocholesterol [37]. Despite endogenous synthesis and the presence of vitamin D in food products, vitamin D deficiencies affect a significant part of the population worldwide [38–40]. Vitamin D is involved in the regulation of calcium-phosphate homeostasis, which is of particular importance for the functioning of the skeletal system [41]. Calcitriol, acting through the vitamin D receptor (VDR), reduces oxidative stress by increasing the level of SODs, GPxs, and GSH expression [33, 42]. Moreover, it was observed that vitamin D reduced the secretion of proinflammatory cytokines, decreasing the level of oxidative stress [43]. The role of vitamin D and its derivatives in cancer is still under investigation. The results of the studies conducted so far are not unequivocal. Some researchers point to a significant role of vitamin D deficiency on cancer mortality, while no effect on morbidity [44–46]. On the contrary, some studies do not link cancer with vitamin D levels [47].

Research indicates a positive correlation between the concentration of vitamin D and osteopontin [48, 49]. Osteopontin is a glycoprotein secreted by osteoblasts and osteoclasts involved in shaping the correct bone structure [50]. The presence of this glycoprotein is not limited to the skeletal system. Osteopontin was found in many tissues and body fluids such as brain astrocytes, kidney, smooth muscle, saliva, and milk [50–52]. Tumor cells of lung, gastric, prostate, ovarian, and colorectal cancer were also found to secrete osteopontin [50]. In the course of neoplastic diseases, an increase in the concentration of osteopontin was observed along with an increase in the level of proinflammatory cytokines [53–55]. Osteopontin was found to be a modulator of the immune response [54]. The relationship between osteopontin and oxidative stress has not been analyzed frequently. The results of the research indicate that the concentration of osteopontin positively correlates with the markers of increased oxidative stress [56–59].

Melatonin (N-acetyl-5-methoxytryptamine) is a hormone synthesized and secreted by pinealocytes in the circadian rhythm [60, 61]. Gastrointestinal tract, lymphocytes, ovaries, skin, and retina are sources of extrapineal melatonin independent of the circadian rhythms [62]. The melatonin molecule contains an indole ring that neutralizes ROS directly [63, 64]. Moreover, research indicates that melatonin decreases the level of ROS by activating the silent information regulator 1 (SIRT1) pathway [65]. Melatonin may also indirectly affect the oxidant–antioxidant balance, stimulating the expression of genes encoding antioxidant enzymes, such as SODs and GPxs [66]. In addition to its direct and indirect action, melatonin inactivates ROS through its metabolites, namely, N1-acetyl-N2-formyl-5-methoxykynuramine (AFMK) and N1-acetyl-5-methoxykynuramine (AMK) [67]. The pleiotropic role of melatonin as endo- and paracrine hormone has been analyzed in carcinogenesis [68, 69]. Scientists indicated an oncostatic role of melatonin in breast, ovarian, prostate, oral, gastric, and colorectal tumors [70]. One of the mechanisms of the oncostatic action of melatonin seems to be based on the reduction of ROS levels [71, 72]. Moreover, melatonin was found to hinder angiogenesis and increase apoptosis of cancer cells [73]. However, little is known about the role of melatonin in LOCP cancer.

So far, only a few studies on the activity of antioxidant enzymes and lipid peroxidation markers in patients with LOCP have been conducted. The results described in the literature are not unequivocal. A decrease in SOD, CAT, and GPx activities with an increase in MDA concentration has been most frequently reported [74–76]. Still, according to some other research, no changes in the activity of antioxidant enzymes in the course of LOCP have been observed [77]. The relationship between the activity of antioxidant enzymes and vitamin D and osteopontin and melatonin has not been studied. Examining the mechanisms related to vitamin D, osteopontin, melatonin, and oxidative stress in the course of LOCP seems to be important for a better understanding of the patophysiology of this type of cancer, as well as for finding new methods of treatment and prevention. Thus, the aim of this study was to determine the activities of selected antioxidant enzymes, as well as the concentrations of vitamin D, osteopontin, melatonin, and MDA in the course of lip, oral cavity, and pharyngeal cancer.

## 2. Materials and Methods

### 2.1. Participants

The study involved 45 patients diagnosed with *carcinoma* in situ of lip, oral cavity, or pharynx according to the International Classification of Diseases–11th Revision (ICD-11)–2E60.0 [78]. The patients were divided into two groups according to their age, namely, younger cancer group (YCG) and older cancer group (OCG). The classification of patients into age groups was based on the United Nations report, which stated that old age begins after the age of 65 [79]. The participants were treated at the Oncology Center, Prof. Franciszek Łukaszczyk Memorial Hospital, Bydgoszcz, Poland. The patients were referred for planning radiotherapy using positron emission tomography–computed tomography (PET/CT) after FDG ([18F]-fluorodeoxyglucose) administration. The patients with G1 squamous cell carcinoma, G2 squamous cell carcinoma, and nonkeratinizing G2 squamous cell carcinoma in histopathological analysis were included in the study. The patients with other grade and type of tumor were excluded from the study. The control group consisted of 25 healthy volunteers. The criteria of exclusion from the control group were associated conditions known to be caused by or to result in oxidative stress or involving disruption of the oxidant-antioxidant equilibrium (cancer, diabetes, cardiovascular, and infectious diseases). A survey was conducted among the people qualified for the study. The questions concerned tobacco addiction and vitamin D supplementation. The characteristics of the study and control groups are presented in Table 1. The study was approved by the Bioethics Committee of the Nicolaus Copernicus University in Toruń functioning at Collegium Medicum in Bydgoszcz, Poland (consent no. KB 221/2018).

### 2.2. Study Design

The patients were eligible for the study on the day of planning for radiotherapy. Blood samples were collected by qualified medical personnel in the morning (between 8:00 AM and 9:00 AM) after overnight fasting from median cubital vein just prior to the administration of the radiopharmaceutical. Every blood sample was collected into two polypropylene tubes. First tube (vol. 6 mL) contained a clotting activator to obtain blood serum, and another tube (vol. 10 mL) was covered with K_2_EDTA to obtain blood plasma. The tubes were immediately transported under reduced temperature condition to the laboratory for centrifugation (6,000 g for 10 min at 4°C). After centrifugation, blood serum and plasma were separated and stored at -80°C for further analysis. The blood morphotic elements remaining after centrifugation were washed three times with a phosphate-buffered saline (PBS) at a ratio of 1 : 3 and each time centrifuged (6,000 g for 10 min at 4°C) to remove leukocytes and thrombocytes. The red blood cells obtained in this method were mixed with the PBS solution to obtain erythrocytic suspension with a 50% hematocrite index.

### 2.3. Biochemical Analysis

The activity of selected antioxidant enzymes was determined in erythrocytic suspension with the use of spectrophotometric methods. Activity of Zn/Cu-superoxide dismutase (SOD-1; EC 1.15.1.1) was assayed according to the Misra and Fridovich method [80]. Analysis was based on the inhibition of adrenaline oxidation to adrenochrome in alkaline solution at 37°C, which induced a change in the absorbance at 480 nm. Activity of SOD-1 was expressed in IU/g Hb. CAT (EC 1.11.1.6) activity was determined with the use of the Beers and Sizer method [81] by measuring the decrease in the absorbance at 240 nm of a solution of hydrogen peroxide decomposed by the enzyme at 37°C. CAT activity was expressed in IU/g Hb. Activity of cytosolic glutathione peroxidase (GPx; EC 1.11.1.9) was assessed using the method of Paglia and Valentine [82]. The principle of the method for measuring GPx activity is based on the ability of the enzyme to reduce hydrogen peroxide with a simultaneous oxidation of GSH as a coenzyme at 37°C, measured at 340 nm. Activity of GPx was expressed in IU/g Hb. Erythrocytic and plasma MDA concentrations were determined with the method of Buege and Aust [83] in the modification of Esterbauer and Cheeseman [84]. The MDA concentration was expressed as the concentration of thiobarbituric acid-reactive substances (TBARS), measured at 532 nm at room temperature. The MDA concentration in erythrocytes was expressed in nmol/g Hb and in blood plasma in nmol/mL. Hemoglobin (Hb) concentration was evaluated using the Drabkin method [85]. Hemoglobin and selected hemoglobin derivatives under the influence of potassium ferricyanide are oxidized to methemoglobin. The absorbance is measured at 540 nm at room temperature.

Serum concentrations of melatonin, vitamin D, and osteopontin were determined with commercially available enzyme immune assay kits. The kits were used accordingly: an enzyme-linked immunosorbent assay kit for melatonin (Cloud-Clone Corp., Houston, TX, USA), a competitive enzyme-linked immunosorbent assay kit for 25(OH)-vitamin D (Immundiagnostik AG, Bensheim, Germany), and a sandwich enzyme-linked immunosorbent assay kit for human osteopontin (BioVendor, Brno, Czech Republic). The measurements were made according to manufacturer's instructions. The enzyme immune assay kits used in the study contain the reagents necessary for the study, standard concentration analytes, blank, and control samples. The principle of the assay is to bind the antigen by specific anti-human monoclonal antibodies that coat the wells of microplates found in the kits. The antigen concentration was determined from the calibration curve. The concentrations of melatonin, vitamin D, and osteopontin were expressed in pg/mL, ng/mL, and nmol/L, respectively.

### 2.4. Statistical Analysis

Statistical analysis was performed using the Statistica 13.3 (TIBCO Software Inc.). The results were presented as means ± S.E.M. Statistical analysis included Student's *t*-test for independent samples, for the comparison of study group and control group, Shapiro-Wilk test to test hypothesis of normal distribution, Levene's test to asses homogeneity of variances. Pearson's correlation coefficient was used to quantify the relationship between the parameters measured. The level of significance was set at *p* < 0.05.

## 3. Results

Anthropometric and clinical characteristic of patients with lip, oral cavity, or pharyngeal cancer and healthy group were presented in Table 1. No significant differences were found between YCG and control group. There was a statistically significant difference in the age of the patients between the YCG, control group, and OCG.

**Figure 1:**
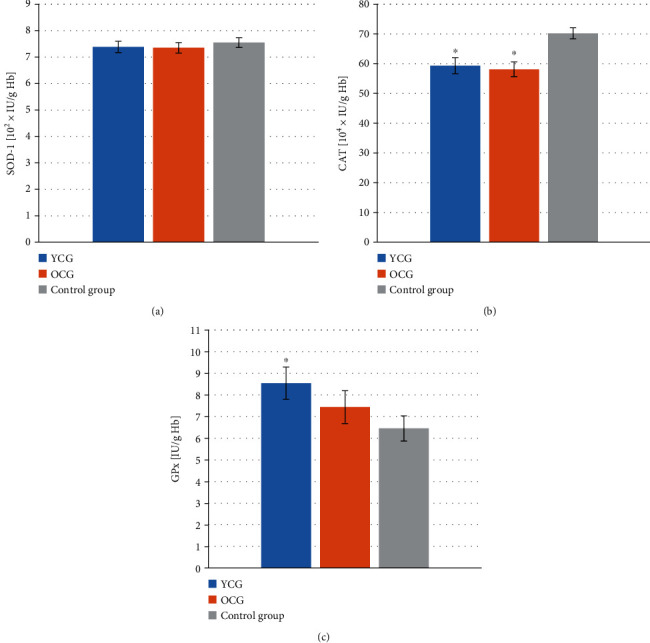
Activity of antioxidant enzymes in the erythrocytes of patients with lip, oral cavity, or pharyngeal cancer depending on age and in the healthy group. (a) Zn/Cu-superoxide dismutase (SOD-1) activity, (b) catalase (CAT) activity, (c) cytosolic glutathione peroxidase (GPx) activity. YCG: younger cancer group—mean age 58.24 ± 1.29 yrs; OCG: older cancer group—mean age 69.70 ± 1.49 yrs. Data are presented as the means ± S.E.M. ^∗^*p* < 0.05 vs. control group.

The SOD-1 activity was similar in all groups and amounted to 738 ± 21 IU/g Hb in YCG, 735 ± 19 IU/g Hb in OCG, and 755 ± 18 IU/g Hb in control group. The statistically lower CAT activity was observed in YCG (59.34 ± 2.6810^4^ × IU/g Hb) and OCG (58.11 ± 2.4710^4^ × IU/g Hb) groups compared to the control group (70.19 ± 1.8710^4^ × IU/g Hb). The activity of GPx in YCG was 8.54 ± 0.75 IU/g Hb and was significantly higher compared to the control group (6.45 ± 0.58 IU/g Hb). In OCG, the mean GPx activity was 7.44 ± 0.76 IU/g Hb. The results concerning the activity of antioxidant enzymes are presented in Figure 1.

**Figure 2:**
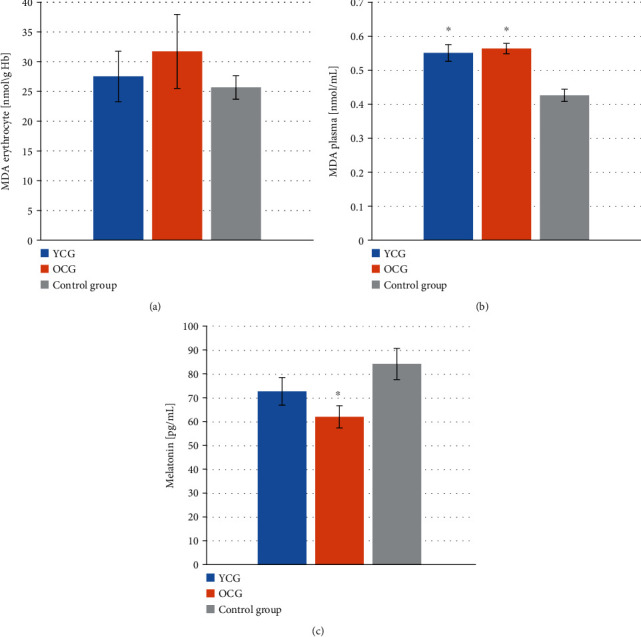
Concentration of malondialdehyde (MDA) and melatonin in patients with lip, oral cavity, or pharyngeal cancer depending on age and in the healthy group. (a) Erythrocytic MDA concentration, (b) plasma MDA concentration, (c) melatonin concentration. YCG: younger cancer group—mean age 58.24 ± 1.29 yrs; OCG: older cancer group—mean age 69.70 ± 1.49 yrs. Data are presented as the means ± S.E.M. ^∗^*p* < 0.05, vs. control group.

There were no statistically significant differences in the concentration of erythrocytic MDA in YCG (27.52 ± 4.23 nmol/g Hb), OCG (31.74 ± 6.21 nmol/g Hb), and control group (25.66 ± 1.98 nmol/g Hb). Significantly higher concentrations of MDA were observed in the plasma of YCG and OCG patients, amounting to 0.55 ± 0.02 and 0.56 ± 0.01 nmol/mL, respectively. In the control group, the plasma MDA level was 0.42 ± 0.02 nmol/mL. The melatonin level in OCG was 62.12 ± 4.70 pg/mL and was significantly lower than in the control group (84.33 ± 6.54 pg/mL). The concentration of melatonin in YCG was 72.83 ± 5.80 pg/mL, and no statistically significant differences were observed in relation to the other study groups.  Figure 2 shows the concentration of MDA and melatonin in the graphs.

**Figure 3:**
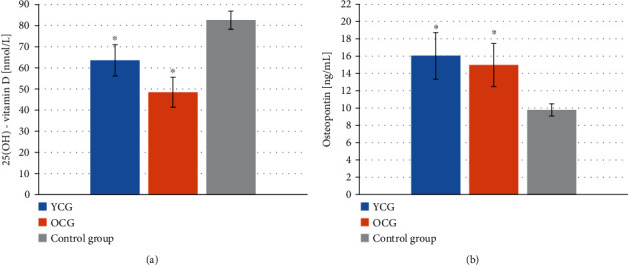
Concentration of 25(OH)-vitamin D and osteopontin in the blood serum of patients with lip, oral cavity, or pharyngeal cancer depending on age and in the healthy group. (a) 25(OH)-vitamin D concentration, (b) osteopontin concentration. YCG: younger cancer group—mean age 58.24 ± 1.29 yrs; OCG: older cancer group—mean age 69.70 ± 1.49 yrs. Data are presented as the means ± S.E.M. ^∗^*p* < 0.05 vs. control group.

The concentration of 25(OH)-vitamin D in the serum of the healthy people was 82.57 ± 4.28 ng/mL. Considerably lower values were observed in YCG and OCG, amounting to 63.55 ± 7.36 ng/mL and 48.42 ± 7.09 ng/mL, respectively. The levels of osteopontin in the LOCP patient groups were significantly higher compared to the healthy group. The concentration of osteopontin in YCG and OCG was 16.04 ± 2.69 nmol/L and 14.98 ± 2.48 nmol/L, respectively, while in the control group, it was 9.78 ± 0.72 nmol/L.  Figure 3 shows the graphs of 25(OH)-vitamin D and osteopontin concentration in the study groups.

**Figure 4:**
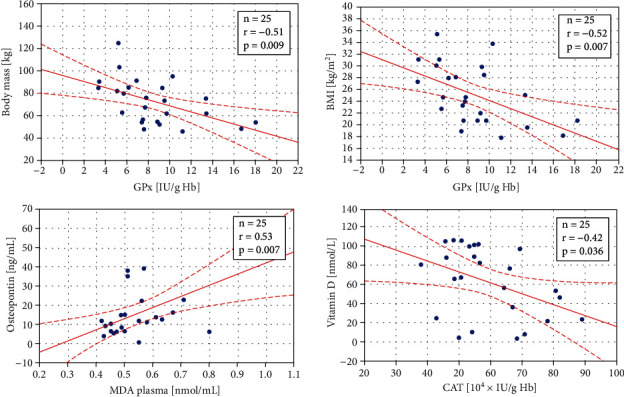
Statistically significant correlations in the younger cancer group (mean age 58.24 ± 1.29 yrs) between body mass and glutathione peroxidase (GPx) activity, body mass index (BMI) and GPx, osteopontin and plasma malondialdehyde (MDA) level, and vitamin D concentration and catalase (CAT) activity. The regression line is marked with a solid line, while the confidence intervals of 0.95 are marked with a dashed line.

**Figure 5:**
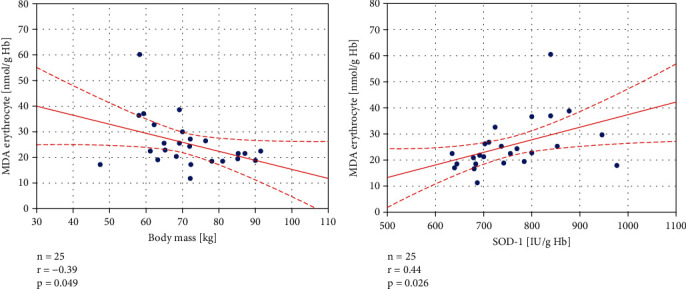
Statistically significant correlations in the healthy, control group between erythrocyte malondialdehyde (MDA) level and body mass, erythrocyte MDA concentration, and Zn/Cu-superoxide dismutase (SOD-1) activity. The regression line is marked with a solid line, while the confidence intervals of 0.95 are marked with a dashed line.

The obtained data were also tested for the presence of correlations. In YCG, statistically significant negative correlations were observed between GPx and body mass (*r* = −0.51, *p* = 0.009), GPx and BMI (*r* = −0.52, *p* = 0.007), and CAT and vitamin D (*r* = −0.42, *p* = 0.036), whereas a significant positive correlation was found between plasma MDA and osteopontin (*r* = 0.53, *p* = 0.007) (see Figure 4). In the control group, a positive correlation between SOD-1 and erythrocytic MDA (*r* = 0.44, *p* = 0.026) and a negative correlation between body mass and erythrocytic MDA (*r* = −0.39, *p* = 0.049) were observed (Figure 5). No statistically significant correlations were found in OCG.

## 4. Discussion

**Figure 6:**
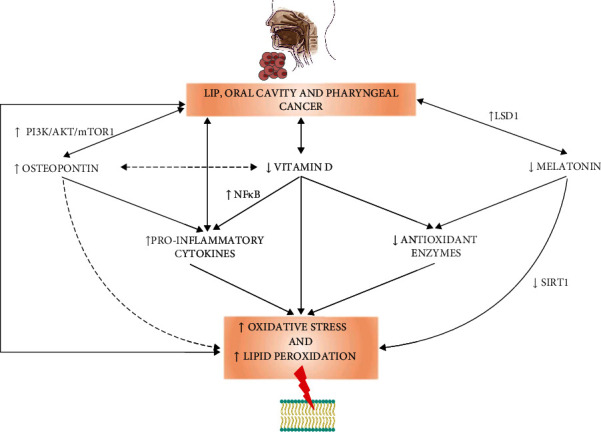
Putative mechanisms linking oxidative stress, antioxidant enzymes, vitamin D, osteopontin, and melatonin in lip, oral cavity, and pharyngeal cancer. Abbreviations used: LSD1: lysine-specific demethylase; NF*κ*B: nuclear factor kappa-light-chain-enhancer of activated B cells; PI3K/AKT/mTOR1: phosphoinositide 3-kinase/protein kinase B/mechanistic target of rapamycin; SIRT1: silent information regulator 1.

As mentioned earlier, LOCP are relatively rare compared to other neoplastic diseases. Due to the small number of patients with such a diagnosis, there are few studies in which the mechanisms of antioxidant defense and the concentration of melatonin, osteopontin, and vitamin D were analyzed [74–77, 86–99].  Figure 6 presents putative mechanisms linking oxidative stress, antioxidant enzymes, vitamin D, osteopontin, and melatonin in LOCP carcinogenesis.

In the present study, specific modifications in the oxidant-antioxidant homeostasis, including a decrease in CAT and increases in GPx (in YCG) and blood plasma MDA, were found in the LOCP patients when compared to the healthy people. Moreover, GPx activity was found to negatively correlate with body mass and BMI in the YCG. Surprisingly, no statistically significant differences in SOD-1 activity were observed. This result is in contrast with the findings of other studies. In the study conducted by Gurudath et al. [86], a decrease in SOD-1 activity in patients with cancer of oral cavity was indicated. The study group consisted of 25 patients with oral cancer. Only smokers and tobacco chewers were included. Average age of the examined subjects was 53 yrs. The control group consisted of 25 healthy people; no information was provided on smoking or chewing tobacco in this group. Also, in the study of Subapriya et al. [87], the activity of SOD in group of 12 patients with oral precancerous lesions or oral cancer was tested. All subjects included in the study were between 35 and 60 yrs old and smoked or chewed tobacco. The cancer patients showed significantly lower SOD activity compared to the control group. Sabitha and Shyamaladevi [76] analyzed 12 blood samples obtained from patients with stage III oral cancer. The authors did not provide the age or information about the addictions of people participating in the study. Also, in that study, a significant decrease in SOD activity was observed in the course of cancer. Similarly, in the research by Manoharan et al. [75] decreased SOD activity was observed. A group of 46 men with oral cancer aged 40 to 60 years old was examined. The control group was free from smoking and chewing tobacco; no such information was provided in the context of the study group. Decreased activity of SOD in red blood cells was also reported by Patel et al. [77]. The age range for oral cancer patients was 22-75 years with a median of 45 years. A group of 126 patients, 113 of whom smoked or chewed tobacco, was tested. Different results were observed in the study of Huo et al. [74]. In their study, a group of 25 patients of both sexes aged 40 to 45 diagnosed with oral squamous cell carcinoma were investigated. Only smokers and tobacco chewers were included in the study. A healthy control group was free of tobacco chewing and smoking habits. The activity of SOD and CAT, as well as the level of erythrocytic MDA, was tested. SOD activity was higher in the group with neoplastic disease. In the case of observed in the present study lower CAT activity in the LOCP patients, similar results were reported by Huo et al. [74], Subapriya et al. [87], Sabitha and Shyamaladevi [76], and Manoharan et al. [75]. On the contrary, Patel et al. [77] did not observe any statistically significant changes in catalase activity in oral cancer patients. In the present study, we noted significantly higher GPx activity in the middle-aged LOCP patients than in the control group. The different results were described by Gurudath et al. [86], Subapriya et al. [87], Sabitha and Shyamaladevi [76], and Manoharan et al. [75]. The authors of these studies observed that GPx activity decreased in the course of cancer. In our study, erythrocytic MDA showed no statistically significant variability between the studied groups, unlike MDA level in blood plasma, which was higher in the cancer patients. In the other studies, increases in the level of MDA in plasma or serum and red blood cells in patients with cancers of the oral cavity and pharynx were unanimously indicated [74–76, 88–90].

Considering the differences between the studies, it is worth noting that the analyzed studies were conducted on small groups of patients of the Asian population (mainly India) [74–77, 86–90]. Additionally, in the study groups, a significant proportion of patients were smokers or chewing tobacco. The relationship between tobacco addiction and ROS generation, which leads to an increase in the level of oxidative stress, was confirmed in numerous studies [100, 101], so this factor could significantly influence the obtained results. The age of the patients is also known to have an impact on the oxidant-antioxidant balance of the organism [102, 103]. The discussed studies were carried out on patients from the age of 40, whereas in the present study, the patients were older [102, 103]. It is worth mentioning that the activity of SOD-1 is dependent on the zinc level in the diet, whereas GPx is an enzyme dependent on selenium. Deficiencies resulting from an unbalanced diet may reduce the activity of SOD-1 and/or GPx. The lower activity of GPx in OCG compared to YCG may be the evidence of selenium deficiency in the diet of the elderly patients with LOCP cancer. Hydrogen peroxide (H_2_O_2_) is a substrate for both CAT and GPxs [104, 105]. In the present study, lower CAT and higher GPx activities were observed in the cancer patients compared to the healthy control group. The lower activity of CAT might be compensated by the increase in GPx activity. Thus, the glutathione-related antioxidant defense seems to be predominant in the patients. In summary, the results of the present study point to the increased ROS generation and reduction of antioxidant defense mechanisms, which are characteristic of neoplastic diseases [18]. Increased levels of lipid peroxidation and MDA could be a consequence of the disturbance of oxidant-antioxidant homeostasis and might be involved in the carcinogenesis.

Research by Liu et al. [91] indicates the important role of melatonin as a ROS scavenger in oral cancer. The research was conducted on human umbilical vein endothelial cells (HUVECs) and six human oral cancer cell lines, including SCC25, SCC9, Tca8113, Cal27, FaDu, and human normal oral keratinocytes (hNOKs). The addition of melatonin (1 mM) to the culture medium significantly reduced the level of ROS in the Cal27 and FaDu cells. Concurrently, melatonin reduced the proliferation and induced the apoptosis of oral cancer cells. Observed inactivation of ROS-reliant Akt signaling significantly decreased the mobility of cancer cells. Inhibition of angiogenesis and reduction in tumor mass were also found. Yang et al. [92] analyzed oral squamous cell carcinoma (OSCC) tissue arrays. The reduction of lysine-specific demethylase (LSD1) expression under the influence of melatonin (0.1 g/mL) was described. Lower LSD-1 expression significantly reduced tumor cell proliferation. Human nasopharyngeal carcinoma (HONE-1), NPC-39, and NPC-BM cell line incubated in a solution containing melatonin (50 ng/mL) were investigated by Ho et al. [93]. Presence of melatonin inhibited TPA-induced cell motility by regulating the matrix metalloproteinase-9 (MMP-9) expression in nasopharyngeal neoplasm cells. Many scientists point to the protective role of melatonin in oral and nasopharyngeal cavity diseases mainly by reducing oxidative stress [106–109]. However, in the present study, a statistically significant lower melatonin concentration in patients with lip, oral cavity, or pharynx cancer compared to the healthy group was only observed in OCG. During the aging, the synthesis and secretion of melatonin are reduced [110]. It could indicate that melatonin deficiency is not particularly involved in the pathogenesis of LOCP [106–109].

Vitamin D deficiency was found to correlate with mortality in the course of neoplastic diseases [44–46]. Calcitriol modulates immune response of the tumor microenvironment through the inactivation of the NF*κ*B (nuclear factor kappa-light-chain-enhancer of activated B cells) pathway [46]. The reference value for vitamin D in blood plasma is 75-125 nmol/L [111]. In our study, we observed that in the group of cancer patients, the vitamin D concentration was below the normal level. It is in accordance with the results of another research. Vitamin D deficiency was found to correlate with mortality in the course of neoplastic diseases [44–46]. The role of vitamin D in oral squamous cell carcinoma was investigated by Verma et al. [94]. Female C57BL/6 mice exposed to 4-nitroquinoline-1-oxide (4NQO) carcinogen were used. The animals were supplemented with vitamin D in the dose of 25-10,000 IU. The inhibition of tumor growth was observed. Beneficial effect in lowering oral mucositis in patients with head and neck cancer was also described by Bakr et al. [95]. The study involved 45 patients treated with radiotherapy divided into three groups. Two groups received topical oral vitamin D gel. Before the intervention, the levels of vitamin D in the blood serum of the patients were found to be deficient. The applied treatment not only reduced oral mucositis but also increased the level of vitamin D in the blood serum of patients. Also, Anand et al. [96] described vitamin D deficiency in oral cavity cancer patients. Moreover, the VDR overexpression in the course of oral cavity neoplasms was indicated in that study. Additionally, vitamin D appeared to play a special role in maintaining oral cavity health [112]. The surprising result of the present study is the negative correlation between CAT activity and vitamin D in the cancer group, suggesting that some regulatory mechanisms might be involved in the course of the disease. Undoubtedly, the role of vitamin D and its deficiency in LOCP cancer requires further research [112].

A significantly higher concentration of osteopontin was observed in our study in the LOCP patients. Also, Jeyasivanesan et al. [97] observed elevated osteopontin levels in patients with oral squamous cell carcinoma. Significant expression of osteopontin in salivary gland tumors was demonstrated in the study by Darling et al. [98]. Muramatsu et al. [99] analysed the effect of osteopontin levels on the invasiveness of oral cavity neoplasms. The study, performed on human oral squamous cell carcinoma cell lines, namely, HSC2, HSC3, HSC4, SAS, KB, and BSC-OF, revealed that high levels of osteopontin may increase the probability of metastasis. However, the mechanisms that link osteopontin and oral carcinomas have been not fully described yet. Presumably, osteopontin binds to integrin A4*β*1 and CD44 receptors, activating phosphoinositide 3-kinase/protein kinase B/mechanistic target of rapamycin (PI3K/AKT/mTOR1) pathway. In next step, mTOR1 regulates estrogen-related receptor alpha (ERR*α*), which binds to DNA and active transcription of osteopontin [113]. Overexpression of osteopontin was associated with increased angiogenesis, cancer cell proliferation, mobility, survival, invasion, and metastasis [113]. The positive correlation between osteopontin and lipid peroxidation, found in the present study, points to the association of the protein with deleterious oxidative processes in plasma membranes. Undoubtedly, these relations should be under further investigation.

The present study has some limitations. The small number of participants is a limiting factor. However, to the best of the authors' knowledge, no study has been conducted with the participation of patients with lip, oral cavity, and pharyngeal cancer of the European population, in which the mechanisms of antioxidant defense and the role of melatonin, vitamin D, and osteopontin were simultaneously analyzed.

## 5. Conclusions

The obtained results indicate a disruption of oxidant-antioxidant homeostasis in the lip, oral cavity, and pharyngeal cancer patients. Impaired antioxidant enzymatic defense and increased lipid peroxidation, correlated with high levels of osteopontin, were determined in this type of cancer. According to the results of the conducted study, melatonin seems not to be involved in the pathogenesis of the analyzed group of neoplasms. However, vitamin D deficiency in the LOCP patients was found. The role of elevated osteopontin in the pathogenesis of lip, oral cavity, and pharyngeal carcinoma requires further research. The simultaneous testing of vitamin D and osteopontin levels seems to be particularly noteworthy.

## Figures and Tables

**Table 1:** Anthropometric and clinical characteristic of patients with lip, oral cavity, or pharyngeal cancer and healthy volunteers (control group). Each value is mean ± S.E.M. YCG: younger cancer group; OCG: older cancer group, ^∗^*p* < 0.05 vs. OCG.

Parameter	YCG	OCG	Control group
*n* (male/female)	25 (15/10)	20 (14/6)	25 (11/14)
Age [yrs]	58.24 ± 1.29^∗^	69.7 ± 1.49	55.36 ± 1.17^∗^
Body mass [kg]	72.53 ± 3.95	71.39 ± 3.52	71.02 ± 2.22
Height [cm]	168.92 ± 1.84	168.55 ± 1.52	169.88 ± 1.72
BMI [kg/m^2^]	25.09 ± 0.99	25.00 ± 1.02	24.50 ± 0.47
Current smoker (y/n)	7/18	5/15	4/21
Vitamin D supplementation (y/n)	5/20	4/16	8/17

## Data Availability

Data are available on request due to privacy/ethical restrictions.
